# The Role of Ayurveda in Prostate Cancer Management

**DOI:** 10.1177/15347354251330906

**Published:** 2025-03-29

**Authors:** Parnian Jabbari, Omid Yazdanpanah, David J. Benjamin, Arash Rezazadeh Kalebasty

**Affiliations:** 1 University of California, Riverside, CA, USA; 2 University of California, Irvine, CA, USA; 3 Hoag Family Cancer Institute, Newport Beach, CA, USA

**Keywords:** prostate cancer, Ayurveda, alternative medicine, clinical trials

## Abstract

Ayurveda is commonly utilized in the treatment of medical ailments but has yet to gain traction in incorporation into allopathic medicine. Prostate cancer is the most common cancer among men and presents a significant public health burden across the globe. Despite advancements in the management of advanced prostate cancer including androgen deprivation therapy and novel hormonal therapies, men may eventually develop resistance to hormonal therapy. As such, there is an urgent need for novel therapeutic options in treating this malignancy. This review examines the pre-clinical evidence for Ayurveda medicinal plants such as *Withania somnifera*, *Glycyrrhiza* spp, *Momordica* spp, *Boswellia*, and *Bacopa monnieri* and their potential application in managing prostate cancer. Several in-vitro and pre-clinical studies suggest potentials for these plants or their derivatives in preventing or treating prostate cancers. Despite strong evidence of efficacy of these plants to potentially improve the outcome of prostate cancer, clinical trials are required to evaluate which plants may be most efficacious and to determine effective dosing strategies, as well as the use of ayurvedic plants as standalone therapies or in combination with conventional prostate cancer treatments.

## Introduction

Maintaining health and longevity has been an age-old concern of mankind. Careful observations led to the discovery of the relationship between certain practices and natural compounds and improved health. These discoveries led to the formation of various systems of medicine across different geographic regions, long before the advent of allopathic medicine.^1^ One such system is Ayurvedic medicine. Originating in India, Ayurveda emphasizes the interplay between the human body’s constitution and natural elements in maintaining health.^2^ The regulatory principles of the body and mind are categorized into 3 doshas: Vata, Pitta, and Kapha. Different practices and elements affect these doshas and can promote or demote their balance.^2^ An important constituent of Ayurveda is the knowledge of how different elements affect these doshas, the knowledge that is compiled in different *Vedas*, particularly describing medicinal plants and how they affect the doshas, hence their use in Ayurvedic medicine. The composition of these doshas in the body (known as Prakriti in Ayurveda) determines how it will respond to each of these medicinal plants.^2^

Even though medicinal plants are currently used for homeopathy, or incorporated into modern diets as an effort for longevity,^3^ medicinal plants are often not incorporated in allopathic medicine.^3^ However, several medications used in allopathic medicine are derived from medicinal plants and their active ingredients. A remarkable example of such medications is artemisinin derived from *Artemisia annua* which was a breakthrough in treatment of malaria, despite later resistance which developed against the drug. Recent preclinical and clinical data suggest that the dried whole plant or infusions of *A. annua* are more effective than artemisinin combination therapy.^4,5^ With the advent of multiomics, new fields of research are dedicated to connecting modern medicine to traditional medicine. As such, Ayurgenomics tries to integrate the Ayurvedic concepts such as Pratriki to allopathic medicine, with the ultimate goal of personalized medicine in mind.^6^

**Figure 1.**
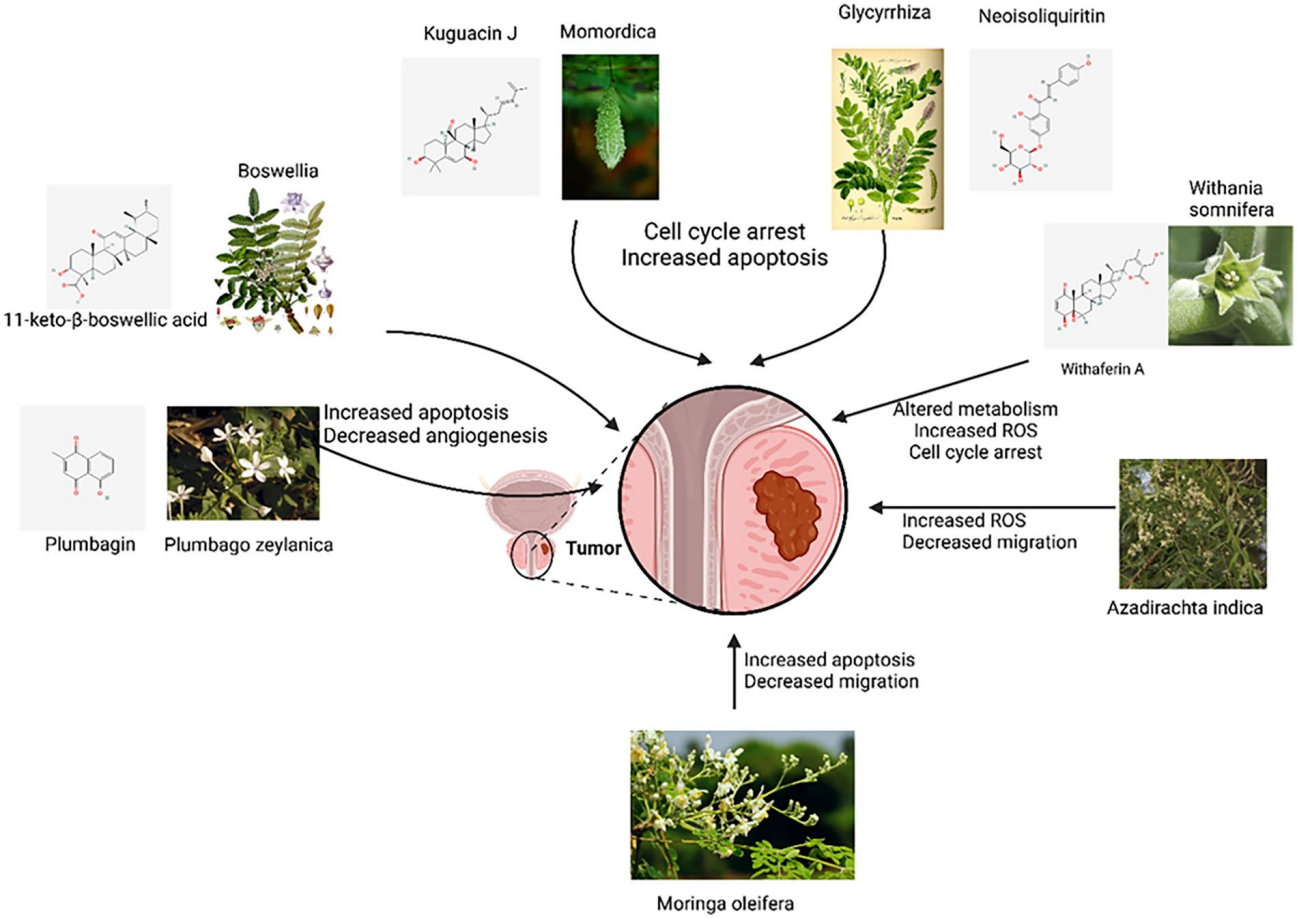
Prominent mechanisms of action of Ayurvedic plants against prostate cancer. Curved arrows show mutual mechanisms. Plant pictures were sourced from Wikipedia. Chemical structures were sourced from PubChem. The figure was created in BioRender.com.

**Figure 2.**
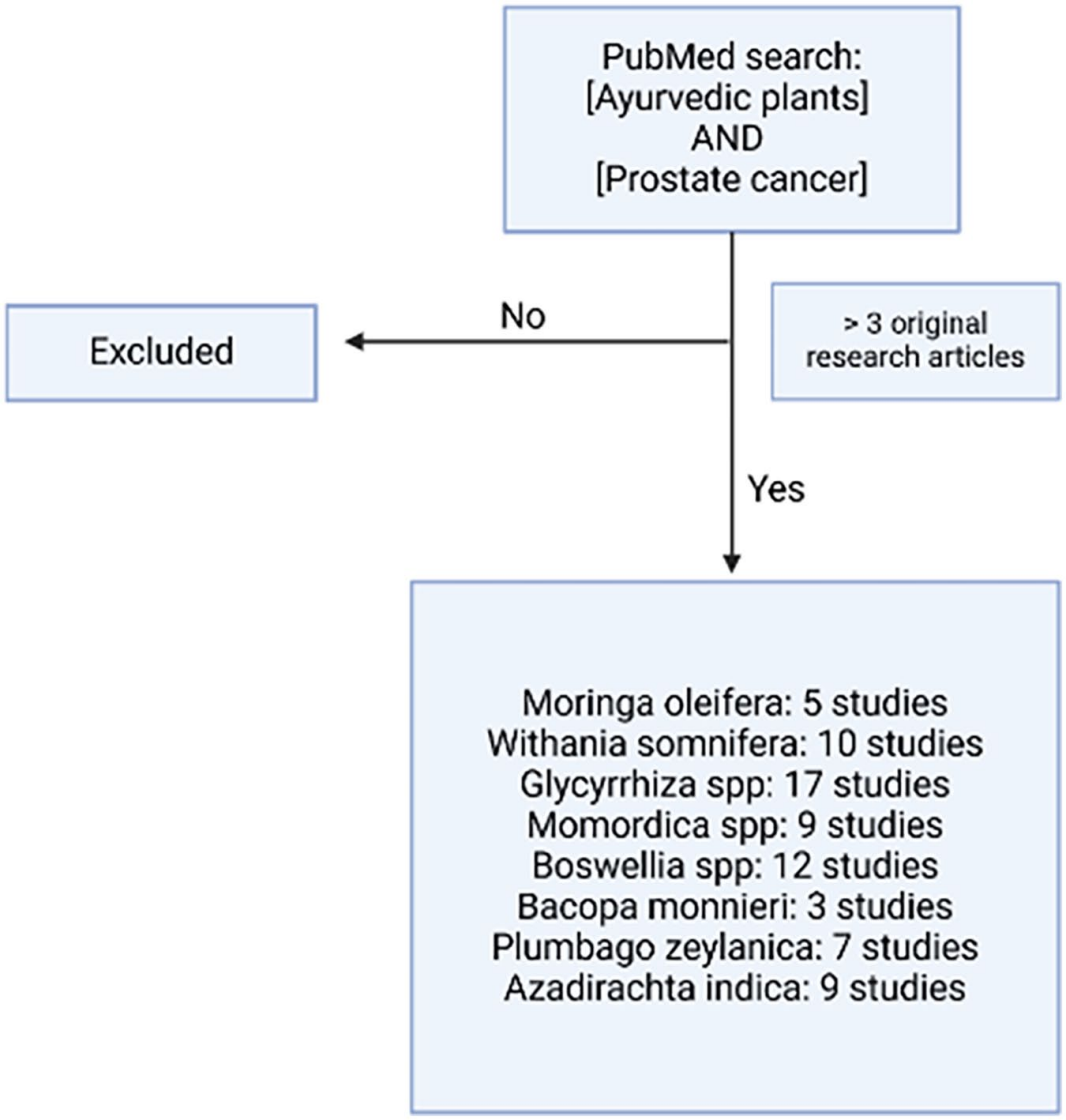
Search terms and inclusion criteria for the study. Plants commonly used in Ayurvedic medicine were search along with [prostate cancer] in PubMed. Plants with 3 or more original articles were included in the study, with the exception of Allium sativum and Curcuma longa.

With the worldwide rise in cancer prevalence associated with the increased life expectancy,^7^ a huge body of research is dedicated to developing effective anticancer medications that can spare healthy cells. Given the effectiveness of these plants in treatment of several diseases, the question that arises is why these plants are not being used as the mainstay or as part of the anti-cancer treatments. Anticarcinogenic active ingredients have been identified in several of these plants and there is a huge body of knowledge regarding their potential use in cancer treatment. Given the prevalence of prostate cancer and its associated mortality rate, herein, we focus on Ayurvedic plants with evidence of potential effectiveness against prostate cancer, their mechanism of action, and the challenges on the way of giving these plants a seat at the anti-cancer table (Figure 1). We conducted a search in October 2023 for original research articles of in vitro, in vivo, or clinical studies published and indexed on PubMed since 2004 for prostate cancer in combination with the common Ayurvedic plants.^8^ Plants with at least 3 published studies were included in this review, including *Withania somnifera*, *Glycyrrhiza* spp, *Momordica* spp, Boswellia, and *Bacopa monnieri* (Figure 2). Turmeric (*Curcuma longa*) and garlic (*Allium sativum*) were excluded from this study due to extensive body of research regarding these plants in the treatment of prostate cancer which was out of scope of this manuscript. In addition, these plants are widely used in different culinary traditions, making them difficult to study in isolation.

## Ayurvedic Plants and Prostate Cancer

### Withania Somnifera

*Withania somnifera*, more commonly known as ashwagandha, is a well-known Ayurvedic plant that balances Vata and Pitta doshas. It is most famous for its rejuvenating, anxiolytic, and memory-improving properties.^9^ However, certain active compounds found in this plant have been shown to be promising for prevention and even treatment of various cancers, including prostate cancer.

In vitro studies have shown that the active compounds in *W. somnifera* can dismantle many of the processes used by prostate cancer cells for tumorigenesis. Active ingredients in *W. somnifera* that are found to have anti-inflammatory properties include alkaloids (anaferine), steroidal lactones (withanolides and withaferines), and saponins.^10^ Among these compounds, withanolides and withaferines are well studied for their effects on cancer cells and tumor microenvironment, including decreased angiogenesis^11^ and altered cytoskeleton structure.^12^

Cancer cells have adapted their metabolism to meet their increased energy demand and assist with their cell signaling.^13,14^ As a result, they switch from oxidative phosphorylation to glycolysis to sustain their ATP pool despite decreased oxygenation of the tumor.^14^ Another mechanism commonly observed in cancer cells is increased levels of fatty acid synthesis, which in turn serves with oncogene palmytoilation.^15^ For this purpose, many cancer cells have increased expression of enzymes required for fatty acid synthesis such as fatty acid synthase (FASN) enzyme complex, ATP-citrate lyase (ACLY), carnitine palmitoyltransferase 1A (CPT1), and acetyl coenzyme A carboxylase 1 (ACC1).^16^ Fatty acid synthesis is an anticancer target and several drugs have been introduced to address increased lipogenesis in cancer cells, however, efficacy, selectivity, and safety are still challenging with the use of these drugs.^17^

A recent in vitro study on androgen-sensitive (LNCaP) and castration-resistant (22RV1) prostate cancer cell lines showed promising anti-lipidogenic properties of *W. somnifera* extract.^18^ In this study, cells were treated with an ethanol extract of *W. somnifera* (WRE) standardized for withaferin A, a withanolide compound found in *W. somnifera*. Cells were treated with different concentrations of withaferin A ranging from 0 to 2 micromolar for 8, 16, and 24 hours. These treatments showed decreased levels of FASN, ACLY, CPTA1, and ACC1 expression and protein levels and the reductions were directly proportional to the dose and duration of treatment with WRE. Furthermore, treatment with WRE showed decreased levels of free fatty acids. Additionally, anti-lipidogenic properties of WRE were compared to that of cerulenin and etomoxir which inhibit FASN and CPT1, respectively. At equimolar concentrations, it was shown that WRE not only decreased fatty acid synthesis more efficiently but also induced apoptosis in both cell lines, an effect not observed with cerulenin or etomoxir. Other studies have unveiled some of the mechanisms through which *W. somnifera* induces apoptosis and affects cell migration in prostatic cancer.

Cancer cells have increased levels of interleukin 8 (IL-8) expression, leading to increased systemic levels of this pro-inflammatory cytokine.^19^ Cancer cells also have increased levels of cyclooxygenase 2 (COX2) which helps them with angiogenesis. Increased levels of these molecules can render prostate cancer cells androgen-independent.^20^ In an in vitro study on androgen-independent prostate cancer cell line PC3 treated with WRE at 10 mcg/mL for 24 hours, it was found that WRE can decrease expression of both IL-8 and COX2, hence potentially decreasing cancer invasiveness.^21^ Similarly, *W. somnifera* was shown to inhibit the activation of nuclear factor kappa B, another important proinflammatory molecule in cancer cells. Even though controversial findings exist regarding the effects of *W. somnifera* on cell cycle and cyclin levels,^22^ a potential mechanism behind the anticancer properties of *W. somnifera* is its lowering effect on various cyclins, including cyclins D1, B1, and A2, which are important checkpoint markers promoting cell cycle progression. Decreased levels of these cyclins in PC3 cells were associated with cell cycle arrest at G2/M phase, unveiling further anticancer mechanisms of *W. somnifera*.^22^ In addition to cell cycle arrest, *W. somnifera* extract can induce apoptosis in prostate cancer cells. In an in vitro study on PC3 prostate cancer cells comparing the effects of lipopolysaccharide-stimulated macrophage products (LSMP) and *W. somnifera* extract on colony formation and apoptosis of cancer cells, it was found that *W. somnifera* could inhibit colony formation of cancer cells, an effect that was absent in LSMP treatment.^23^ Cellular apoptosis observed with *W. somnifera* extract can be attributed to the upregulation of proapoptotic genes including caspases 3, 8, and 9, and the downregulation of antiapoptotic genes such as peroxisome proliferator-activated receptors (PPAR) and cellular FLICE (FADD-like IL-1β-converting enzyme)-inhibitory protein (cFLIP).^24^ Similarly, *W. somnifera* extract, unlike LSMP, reduced the migration of PC3 cells. In addition to regulating cellular pathways, *W. somnifera* is shown to increase the production of reactive oxygen species (ROS) specific to cancer cells as opposed to healthy cells, a mechanism important for the induction of cell cycle arrest and apoptosis in cancer cells.^24^ This specificity of ROS formation to cancer cells is important, as increased ROS production is the mainstay of several chemotherapeutic drugs that other compounds should not oppose.

Despite the several mechanisms mentioned in modulating the anticancer effects of *W. somnifera*, it is important to identify the dosage and conditions in which these effects are observed. An in vitro study found that *W. somnifera* induced the expression of c-Myc and the PI3K pathway, which can have carcinogenic effects.^25^ Furthermore, there may be differences in in vitro and in vivo settings regarding the anticancer effects of this plant. In a double-blind randomized clinical trial in healthy adult males aged 40 to 70 years, it was found that *W. somnifera* increased androgen levels.^26^ Whether these effects are observed in patients with prostate cancer needs to be determined through further clinical trials. Additionally, *W. somnifera* is an inducer of CYP3A4 which is involved in metabolism of several standard treatments for prostate cancer, such as relugolix.^27^ How the combination of *W. somnifera* and conventional prostate cancer treatments can affect the response to treatment needs to be determined at different levels, including through clinical studies.

### Glycyrrhiza spp

*Glycyrrhiza* species, commonly known as licorice, are perennial herbs of which the roots and rhizomes were used as natural sweeteners and in various traditional medicine practices, including Ayurvedic medicine.^28^ Licorice balances Vata and Pitta doshas and improves Kapha dosha. Traditionally, it was used to treat various diseases, including lung, kidney, and liver disease. Currently, licorice supplements are available over the counter for reducing inflammation, cough, improving liver health, helping with menopause symptoms, and digestive problems.^29^ Several studies have found anticancer properties for different *Glycyrrhiza* extracts,^30^ suggesting this plant to have promising effects for treatment of prostate cancer.

*Glycyrrhiza* exerts its anticancer effects on prostate cancer through various mechanisms. Flavonoids found in *Glycyrrhiza* can inhibit cell cycle progression and induce apoptosis in prostate cancer cells. In an in vitro study, treatment of LNCaP and PC3 cell lines with neoisoliquiritin (NEO), a flavonoid found in *Glycyrrhiza*, was shown to induce G0/G1 cell cycle arrest in LNCaP cells and inhibited cell proliferation in a dose- and time-dependent manner through downregulation of cyclin D1 and cyclin-dependent kinase (CDK) 4.^31^ Additionally, transcriptomic comparison of NEO-treated cells with controls suggested androgen-receptor signaling to be affected by NEO treatment in LNCaP cells, and androgen receptor expression was shown to be downregulated by NEO treatment. NEO treatment also inhibited xenograft prostate tumor development in SCID mice, suggesting in vivo effects of the compound as well.^31^ Similar findings were found with the treatment of PC3 and 22RV1 cell lines with isoliquiritigenin.^32^ Isoliquiritigenin treatment for 48 hours showed a dose-dependent decrease in cell viability in these cell lines and induced cell cycle arrest at both G0/G1 and G2/M phase associated with decreased levels of CDK1 and CDC25B and increased expression of cyclin B1, GADD45A, promoting cell apoptosis.^32^ Similar results were observed with the treatment of prostate carcinoma cell line DU-145 with lupiwighteone (Lup), another flavonoid compound found in glycyrrhiza.^33^ Lup treatment of DU-145 cells was shown to induce cell cycle arrest, apoptosis, loss of mitochondrial membrane potential, as well as increased production of ROS in a time- and dose-dependent manner.^33^ In addition to inducing apoptosis, licochalcone-A, another flavonoid compound found in licorice can also induce autophagy in LNCaP cells, in addition to reducing Bcl-2 and inhibiting mammalian target of rapamycin pathway.^34^ Similar patterns of inhibiting cell proliferation and induction of DNA damage have been observed in other in vitro studies using different prostate cancer cell lines, emphasizing the potential of *Glycyrrhiza* in inhibiting prostate cancer cell growth.

Despite promising in vitro effects, caution must be taken when generalizing the anticancer properties of licorice to in vivo effects, due to complexity of the system. In humans, licorice is metabolized to glycyrrhetic acid by intestinal bacteria.^35^ This metabolite is hundreds-fold more potent than glycyrrhiza in inhibiting 11-ß-hydroxysteroid dehydrogenase, which leads to accumulation of cortisol and its mineralocorticoid effects. Even though oral bioavailability of licorice is low, prolonged consumption of licorice in patients under standard treatment for prostate cancer, such as with abiraterone and enzalutamide which cause hypertension, may lead to worsening of adverse effects associated with both licorice and the standard treatment.^36,37^ In addition, licorice can induce hepatic CYP450 enzymes which also metabolize several of the chemotherapy medications, such as docetaxel. This interaction can decrease the effective dose of the medication. Additional clinical research is warranted to understand the interactions between conventional prostate cancer medications and licorice to identify potentially appropriate dosing.

### Momordica spp

*Momordica* species, commonly known as bitter melon, are tridoshic plants used for their anti-inflammatory, anti-diabetic, and antioxidant properties. These plants are high in anticancer compounds such as flavonoids, triterpenoids, and saponins. Two of the species in this genus, *Momordica charantia* and *Momordica cochinchinesis*, have gained attention for promising anticancer properties found based on their efficacy in reducing P-glycoprotein multidrug resistance and induction of caspase-independent apoptosis and cell cycle arrest in different cancer cells ^38,39^. Many of these anticancer properties observed in *Momordica* species are attributed to Kuguacin J, a cucurbitane-type triterpenoid secondary metabolite, and other kuguaglycosides found in this plant.

In an in vitro study on LNCaP prostate cancer cells,^40^ cells were treated with different concentrations of Kuguacin J (KuJ) isolated from *Momordica charantia* leaf for 24 hours or 48 hours. Cell survival analysis showed a dose- and time-dependent inhibitory effect of KuJ treatment on cell survival associated with increased cleavage of caspases and induction of apoptosis. Cell cycle progression was also affected by decreasing levels of cyclins and CDKs, and induced cell cycle arrest at the G1 phase. In addition, p53 tumor suppressor levels were increased in a dose-dependent manner, and higher doses of KuJ treatment were associated with decreased androgen receptor and prostate-specific antigen expression in these cells.

In another in vitro study, a PC3 cell line was used to determine the mechanisms behind the anticancer properties of *Momordica cochinchinesis* stem extract (MCSE).^41^ Treatment of PC3 cells with MCSE was shown to affect mitochondrial membrane potential and activate caspase complexes while decreasing anti-apoptotic proteins such as Bcl2, leading to apoptosis of cells. Interestingly, these effects were more pronounced in cancer cells due to high IC50 concentration for non-cancerous cells. Similar effects have been observed in treatment of PC-3 cell line with *Momordica* leaf extract.^42^

In another in vitro study,^43^ different preparations of the aril of *Momordica cochinchinesis* were made, and their effect on prostate cancer cells PC3 and LNCaP was evaluated. Of the various preparations made, sonicated extract of the plant was shown to have antiproliferative effects on both cell lines, with more prominent effects on PC3 cells, however, these effects were not dose-dependent. Additionally, treatment with *Momordica* extract was shown to induce cell cycle arrest, especially at the G1 phase, and decreased cell migration associated with decreased matrix metalloproteinase 9 (MMP9) and increased tissue inhibitor of metalloproteinase (TIMP1), indicating the potential of this plant in inhibiting metastasis of cancer cells.

### Boswellia

The genus *Boswellia*, also known as frankincense, are tridoshic plants commonly used for their anti-inflammatory and rejuvenating properties.^44,45^ Studies have found anticancer properties for Boswellia and Boswellia extract that is mostly attributed to 11-keto-β-boswellic acid (AKBA), a pentacyclic triterpenic acid found in them.^46^ Two of the species in this genus, *Boswellia serrata* and *Boswellia carterii*, have gained attention for their inhibitory effects on prostate cancer cells.

Treatment of docetaxel-resistant prostate cancer cells (PC3/Doc) with AKBA isolated from *Boswellia* showed dose-dependent inhibition of proliferation and induction of apoptosis in these cells.^47^ These inhibitory effects on cancer cells were associated with decreased levels of Akt and STAT3 and inhibition of their signaling pathway. Also, AKBA treatment was associated with increased poly-ADP ribose polymerase cleavage and decreased Bcl2 levels in a dose-dependent manner which indicates increased apoptosis of cancer cells.^47^ In vitro treatment of PC3 cells with ac-LA showed increased apoptosis of these cells associated with inhibition of Akt and mTOR signaling and disturbed mitochondrial membrane potential.^48^ Ac-LA treatment also decreased vascular-endothelial growth factor (VEGF) production of PC3 cells, which indicates inhibiting angiogenesis in tumors. Topical treatment of xenograft PC3 cells in chick chorioallantoic membranes or in nude mice showed decreased growth of implanted cancer cells in vivo, accompanied by decreased angiogenesis. These inhibitory effects were more prominent in treatment with ac-LA than with lupeol, and inhibitory effects of ac-LA were comparable to those of docetaxel.^48^ Similar inhibitory effects on Akt and mTOR signaling were also observed in treatment of androgen-independent prostate cancer cells treated with α-acetoxy-tirucallic acid αATA (8,24).^49^ This treatment was associated with increased apoptosis both in vitro and in vivo xenografts of the cancer cells, without significant toxicity to normal cells.^49^ Boswellic acid can also increase antioxidant response. In vivo treatment of rats with boswellic acid showed increased activity of antioxidant enzymes superoxide dismutase and catalase. Such effects can protect the testicular cells against environmental stressors.^50^

However, further work is warranted in translating these findings to clinical levels. *Boswellia* extract is found to inhibit CYP3A4 and CYP2C9,^51^ which may interfere with conventional prostate cancer treatments. In addition, some plants from this genus, such as *Boswellia sacra*, are shown to increase testosterone levels in animal models which can interfere with androgen deprivation therapy for androgen-sensitive prostate cancer.^52^ It is important to investigate whether such androgen-boosting effects are observed only in healthy individuals before their clinical application. Further, the increase in antioxidant response observed in treatment with boswellic acid may interfere with chemotherapeutic agents that deliver their effects through oxidative stress on cancer cells. These potential interferences with conventional cancer treatments need to be investigated in preclinical and clinical trials.

### Bacopa monnieri

*Bacopa monnieri*, more commonly known as Brahmi, is a well-studied Ayurvedic plant for balancing the pitta dosha. It is commonly used for its neuroprotective, anti-inflammatory, and antioxidant effects. Recent studies have shown promising anticancer effects of Brahmi in different cancer types, including breast, colon, liver, and prostate cancer.^53^ Even though research on the effects of this plant on prostate cancer is sparse, an in vitro study has shown promising effects. Treatment of prostate cancer cell line DU-145 with *Bacopa monnieri* extract derived from artificial digestive juices showed a dose-dependent inhibitory effect of this plant on the survival of DU-145 cancer cells. In addition, this treatment showed decreased motility of cancer cells, indicating its potential for addressing the invasiveness of cancer cells. Similar to other Ayurvedic plants, *Bacopa monnieri* is also an inhibitor of several CYP450 enzymes, potentially affecting the response to treatment when taken in combination with conventional prostate cancer medications.^54^

### Plumbago zeylanica

*Plumbago zeylanica*, also known as leadwort, has a wide range of uses in Ayurvedic medicine such as anti-inflammation, anti-cancer, and wound healing to name a few^55^ attributed to its various derivatives including flavonoids, alkaloids, triterpenoids, and napthoquinones.

Napthoquinones have been studied for their anti-cancer properties against several cancers, including prostate cancer.^56,57^ They can inhibit growth and induce apoptosis in cancer cells.^58^ In a xenograft model of PC-3M-luciferase prostate cancer cells, treatment with Plumbagin, a quinoid isolated from the roots of *Plumbago zeylanica*, inhibited tumor growth and decreased metastasis^57^ associated with decreased levels of protein kinase C, survivin, Bcl, different MMPs, and STAT3 phosphorylation along with induction of inducible nitric oxide synthase. Plumbagin treatment in mice also showed decreased expression of VEGF and CD31, indicating lower levels of angiogenesis in tumors. In vitro studies on treatment of prostate cancer cell lines with Plumbagin showed increased apoptosis in these cells due to increased levels of reactive oxygen species.^59,60^ While toxicity of crude extractions of Plumbagin in normal cells may be a concern, nanoparticle preparations of Plumbagin showed to decrease potential toxicity of Plumbagin to normal cells while exerting anti-cancer effects including decreased migration^61^ and induction of apoptosis^62^ in cancer cells in a dose-dependent manner.

Oral administration of plumbagins in mice has shown to affect CYP450 levels along with increasing aspartate aminotransferase and alanine transaminase, potentially interacting with other drugs used to treat prostate cancer.^63,64^

### Azadirachta indica

*Azadirachta indica*, also known as Neem tree, has been widely used in traditional medicine including Ayurvedic medicine for its antioxidant properties attributable to its various active compounds such as azadirachtin, nimbolinin, nimbin, and salannin.^65^ In vitro and in vivo evidence suggests anti-cancer properties of this plant against several cancers, including prostate cancer, by targeting multiple elements of cancer cells.^66^

Treatment of PC-3 and LNCaP prostate cancer cell lines with ethanolic neem leaf extract showed activation of apoptotic pathways including Bcl-2, cytochrome C, and caspase 3 expression.^67^ In addition, neem leaf extract inhibited the PI3K/Akt pathway in both cancer cells lines.^67^ Treatment of PC-3 cell line with neem tree extract also showed increased DNA fragmentation leading to apoptosis.^68^ In vitro studies of treatment of DU145 and LNCaP prostate cancer cell lines with nimbolide, a triterpene compound found in neem tree, showed a dose- and time-dependent decrease in cell viability, migration, and invasion^69^ attributable to inhibition of JAK/STAT signaling and increased production of ROS in these cells. Treatment of mice with transgenic prostate adenocarcinoma led to decreased formation of neoplasia in mice as well as decreased incidence of progression from neoplasia to prostate cancer.^69^ Systemic treatment of mice with established prostate cancer also showed decreased tumor growth and progression of cancer^69^ without significant adverse effects showing promising effects for treatment of prostate cancer.

While studies suggest neem leaf extracts to be safe or associated with minor side effects,^70^ these drugs can increase effectiveness of chemotherapy medications.^71^

### Moringa oleifera

*Moringa* is one of the widely used plants in Ayurvedic and other traditional medicines known for its anti-inflammatory, wound healing and anti-cancer properties. It has recently been appreciated as a superfood to address hypertension, obesity, and other health concerns.^72^

In vitro treatment of prostate cancer cell lines with *Moringa* leaf and seed extracts show promising effects as therapeutic options. Treatment of PC-3 cell line with methanolic *Moringa* leaf extract at different concentrations showed a dose-dependent decline in cell viability in cancer cells, whereas treatment of normal (HEK-293) cells did not show significant cytotoxicity even at doses as high 640 µg/mL.^73^ *Moringa* leaf extract induced apoptosis and increased intracellular ROS and decreased mitochondrial membrane potential in these cells in a dose-dependent manner. Glucomoringin isothiocyanate extracted from *Moringa* seed was used to treat the PC-3 cell line and showed increased apoptosis and cell cycle arrest in a time-dependent manner.^74^ Similar observations were made with treatment of PC-3 cells with *Moringa* alkaloids where cell migration and proliferation were decreased in a dose-dependent manner.^75^ In addition, systemic treatment of mice with PC-3 xenograft with *Moringa* alkaloids led to inhibited growth of cancer cells in vivo.^75^ These effects of *Moringa* extract on cancer cells can be attributed to suppression of the Wnt/β-catenin, Bcl-2, and Hedgehog signaling as well as induction of caspases, Bax, and P53 signaling.

Similar to several other plants, *Moringa* can affect other cancer treatments by inhibiting CYP450 enzymes, which needs to be taken into consideration when used along with other anti-cancer strategies.

## Discussion

Prostate cancer is most common cancer in males with high mortality rates in the metastatic setting.^76^ Current standard of care for prostate cancer includes androgen deprivation therapy and chemotherapy, surgery, and radiation.^77^ However, most prostate cancers progress to become androgen-independent,^78^ which can challenge treatments and leaves chemotherapy as the main treatment option,^79^ and the search for effective, yet less toxic medications is still ongoing.

Use of medicinal plants is common worldwide, with 80% of world’s population using medicinal plants for self-treatment,^80^ and these numbers are growing as there is an increasing trend to use medicinal plants.^81^ However, there are variations in popularity of their use in different geographic regions. Use of medicinal plants and complementary medicine is also common among individuals with cancer,^82^ and Ayurvedic treatment is one of the most commonly utilized treatments particularly in North India.^83,84^ Popularity of these practices can be attributed to a variety of reasons, including improving the ability to fight cancer, emotional and physical well-being, and a sense of control over their health.^82^ Individuals with cancer receive recommendations for these alternative therapies mostly from their families and friends, rather than their healthcare providers.^82^ Many patients do not disclose use of these alternative practices to their physicians for various reasons, and this can result in interactions between the herbal preparations and chemotherapeutic drugs which may not be desirable.^85^

Given the efficacy of Ayurvedic plants based on the in vitro and in vivo studies and their popularity in various regions of the world, it is important to conduct clinical trials on the effects of these plants on cancer patients. These plants have shown promising effects in these studies and they can dismantle several mechanisms and pathways employed by cancer cells and their effect can spare healthy cells, properties that are not easily achieved by single chemotherapy agents. Moreover, these plants can provide cost-effective and easy-to-access treatment options that may be more agreeable to patients due to their natural sourcing. However, a current lack of clinical data prevents physicians and clinicians from making recommendations to patients, while patients may continue to use these plants that may potentially interfere with their treatment.

There are several challenges that need to be addressed before clinical evidence is available to support the use of these Ayurvedic plants as stand-alone or adjuvants for cancer therapeutics. As each of these plants has several active ingredients with their specific anti-cancer properties, to ensure a standardized treatment these plants need to be purified, and various active ingredients need to be isolated. Chemical preparation of these active ingredients is another consideration. Several of these active ingredients such as Withaferin A, Plumbagin, and Moringa, have low water solubility and need to be prepared in organic solvents or delivered as other preparations^86^ which challenges their clinical application. Another important consideration when it comes to use of these plants in the treatment of prostate cancer is their effect on androgen levels. Among the plants reviewed, Ashwagandha^26^ can increase testosterone levels, and Neem tree and Boswellia can decrease testosterone levels^87,88^ and licorice can inhibit androgen receptors.^31^ However, the effects of many Ayurvedic plants such as *Moringa* on testosterone is a subject of debate^89,90^ or have not been extensively studied. Another consideration for use of these plants in treatment of cancers is their acceptability in different cultures. While plants such as Ashwagandha have made their way to the supplement shelves, other plants may not be widely known, which can affect the level of efforts put into research on their use for treatment of prostate cancer.

## Conclusion

Similar to any other agent to be used in humans to address any disorder, these Ayurvedic plants also need to be tested in vivo and through clinical trials to identify the most efficacious plants and also to understand dosing strategies. This clinical data is required on the efficacy of these plants in treating cancers alone or in combination with other standard-of-care drugs. Evidence from such clinical trials is much needed, as either in case of favorable results or unfavorable outcomes, evidence-based decisions can be made by both healthcare providers and patients.
